# Special Issue: Autoimmune Disease Genetics

**DOI:** 10.3390/genes12121937

**Published:** 2021-11-30

**Authors:** Malgorzata Gabriela Wasniewska, Artur Bossowski

**Affiliations:** 1 Pediatric Unit, Department of Human Pathology in Adulthood and Childhood, University of Messina, 98122 Messina, Italy; 2 Endocrinology and Diabetes with a Cardiology Unit, Department of Pediatrics, Medical University of Bialystok, 15-001 Bialystok, Poland; artur.bossowski@umb.edu.pl

Autoimmune diseases (ADs) are characterized by a multifactorial etiology, in which genetic and environmental factors are responsible for the loss of immunological tolerance.

Although the pathogenic mechanism of ADs is still under investigation, the evidence in favor of a genetic basis for ADs is abundant. The different genetic factors could be associated not only with disease susceptibility but also with specific autoantibodies and disease phenotypes. Knowledge of new aspects of ADs could help us to better understand disease etiology and treatment responses and also contribute to the development of new therapy strategies.

The aim of this research topic was to report the most updated views on genetics of autoimmune diseases, with particular attention to organ-specific ones on comorbidities in ADs and their interrelations.

This Special Issue put together five original articles, five reviews (four narrative and one systematic) and one very particular case report, summarizing current knowledge on the epidemiology, pathgenesis (related to genetic susceptibility, chromosomal differences or epigenetics), clinical long-term experience, clustering and interrelation with other ADs, as well as on the peculiarity of ADs in genetic syndromes. 

Five very innovative original research studies on genetic basis and predisposition for AD development have been published on this research topic. Cho et al. described two polymorphisms in GPR174 and ITM2A genes on the X chromosome that might be associated with autoimmune thyroid diseases (AITDs) in Korean children [1]. Naderi et al. identified 17 potential genes proven to be pivotal in psoriasis for therapeutic/diagnostics purposes in that pathology [2].

Zou et al. tried to explain that better knowledge of genetic pathomechanisms, using the disease-associated risk gene transcriptional regulation network, could help prevent any therapeutic effect in the ADs [3]. The experimental study of Starosz et al. shed new light on the role of dendritic cells in the pathomechanism of the pediatric Grave’s disease [4].

The results of the original study of Almeida et al. suggested a cumulative effect of SNPs at the DHCR7, GC, CYP2R1, and CYP24A1 loci on the susceptibility to type 1 diabetes, due to the roles of these genes in the vitamin D metabolic pathway [5].

In the context of this research topic, there are also five reviews that allowed us to increase our pathogenetic and clinical knowledge based on the wide experience of the authors. 

The systematic review *Hashimoto’s Thyroiditis and Grave’s Disease in Genetic Syndromes in Pediatric Age* by Casto et al. confirmed that AITDs show peculiar phenotypic patterns when they occur in association with some genetic disorders, especially chromosomopathies [6]. Turner, Down, Klinefelter, Prader–Willi, Williams, Noonan syndromes, neurofibromatosis type 1, and 22q11.2 and 18q deletion syndromes were evaluated. The authors suggested an accurate screening and monitoring of thyroid function and autoimmunity to improve clinical practice and healthcare in children and adolescents with genetic syndromes. In the narrative reviews, Ferrari and Stagi reported the current literature regarding the genetic, immune, and environmental factors as the possible underlying mechanism of autoimmunity in individuals with Down syndrome [7]; Ben-Skowronek presented an update on IPEX syndrome making the knowledge of the genetics fundamental to introducing novel treatment methods in that pathology [8]; Roszkowska et al. presented actual knowledge relative to the pathogenesis of Sjogren’s Syndrome, considering the role of innate immunity, adaptive immunity, and genetics [9]; and finally, Cucinotta et al. presented an updated overview on the available evidence concerning the etiology (with genetic pattern), pathogenesis, and clinical presentation of pancreatic involvement in pediatric inflammatory bowel diseases [10].

Our Special Issue ends with the description of two siblings, with the same mutations in the AIRE gene associated with two very different phenotypes by Caprino and collaborators [11]. As autoimmune polyendocrinopathy-candidiasis-ectodermal dystrophy (APECED) is a monogenic disease, genetic, epigenetic, and environmental factors might influence the phenotypic expression, although their exact role remains to be elucidated until now.

In conclusion, this research topic provides an important and updated contribution to the subject of autoimmune disease genetics. Several papers highlighted the need for further and prospective studies to clarify certain pathogenetic, clinical, and also therapeutic aspects of ADs.

Finally, ADs are confirmed as an active and still growing area of research in pediatric and adult age. Increasing knowledge of genetic aspects and pathogenetic mechanisms will allow to better determine the predisposition to AD development, to diagnose organs and systems involved dysfunction earlier, and to improve treatment using immunogenetic therapy.
